# Probing technoscience

**DOI:** 10.1007/s10202-011-0103-0

**Published:** 2011-11-30

**Authors:** Karen Kastenhofer, Astrid Schwarz

**Affiliations:** 1 Institute of Technology Assessment, Austrian Academy of Sciences, Strohgasse 45/5, 1030 Wien, Austria; 2 Department of Philosophy, TU Darmstadt, Schloss, 64283 Darmstadt, Germany

In the last three decades, the attention of sociologists, historians and philosophers of science was more and more attracted by the concept “technoscience.” It emphasizes an entanglement of science and technology and it was mainly raised to distinguish a “new” type of scientific activities from “traditional” ones with a different epistemic interest producing different objects with a different ontological status. There is some agreement that it was the Belgian philosopher Hottois (1984) who introduced the term “technoscience.” He used it to refer to a type of science that is done in a technological milieu and that is technology-driven. About a decade later, the philosopher and anthropologist of science, Latour, deployed the term in his seminal work on “science in action” (Latour 1987) to characterize the entangling and disentangling of practices, people, objects and methodologies in scientific activities. The cultural theorist Haraway made technoscience one of her central concepts (e.g. Haraway 1990, 1997), albeit again taking a different direction. In her analysis of the relationship between nature, technology and culture within a technoscience era, she emphasizes the hybrid character of objects in the real-world, and identifies a collapse of traditional dichotomies such as nature and culture, machines and humans, or of the sexes.

During the past decade, an increasing number of scholars have begun to adopt the concept of technoscience, drawing on Latour and Haraway as well as on other literature. An important theoretical input to the formation and conception of technoscience was added by the so-called practical turn, focusing on the epistemic cultures in the laboratory sciences but also in the field sciences. This refers to analyses and research on epistemic cultures initiated by Hacking (1983), Pickering (1992), Knorr Cetina (1999) and Rheinberger (1997, 2006). Technoscience has thus been discussed as a theoretical concept within STS (Science and Technology Studies) as well as an epistemic approach within science (Kastenhofer 2007). Since a few years, the concept is systematically reconsidered and scrutinized from a philosophical and historical perspective (Bensaude-Vincent et al. 2011; Forman 2007; Nordmann 2006; Schwarz and Nordmann 2010). In discussing technoscience, all of these authors focus on the cultural and material dimension of technoscience, especially within the everyday perceptions and practices prevalent either within science or, more generally, within western society. Some—but by far not all—authors also point at differences between what they call “technoscience” on the one hand and traditional science (or traditional technology) on the other.

When trying to apply the term as an analytical tool to different empirical contexts and comparing its various usages, overarching questions about technoscience arise: are the emerging technosciences different from traditional sciences? Do they imply a new relationship between science and technology? Do they perhaps suggest different modes of convergence between these two realms? Or does the concept of technoscience mainly represent an alternative analytical background to be applied to *all* scientific fields alike? What would be the advantage or motivation for such a general shift? What does the label “technoscience” bring to light and what does it obscure? What are the societal implications and governance issues raised by the concept of technoscience? Is it possible to build upon and further develop the concept of epistemic cultures against the background of technoscience studies?

Whereas a previous issue of Poiesis and Praxis (2010, issue 7) has been dedicated to evaluating the relevance of the concept of technoscience for technology assessment and its political dimensions, it is the main aim of this special issue to probe the concept of technoscience in empirical as well as theoretical terms. Thereby, technoscience is very generally understood as pointing at a (proposed) convergence of science and technology, of representing and intervening, of understanding and performing and/or of the natural and the artificial. The individual contributions to this issue result from a special track on “Probing Technoscience” organized at the 2010 conference of the European Association for the Science and Technology in Trento, Italy. They aim at scrutinizing the general conception of technoscience from diverse points of view. They present empirical analyses of emerging technosciences (e.g. nanotechnology, biomedicine, systems biology and synthetic biology) and reflect on the significance of the concept of technoscience within science and technology studies as well as science and technology governance and—more generally—society.

From a sociological perspective, *Peter Wehling*’s account probes the idea of a “technoscientization” that has been postulated for biomedicine and health-care during the past decade by Clark and other sociologists of medicine (Clark et al. 2003). Drawing on material from an empirical study of rare disease patient organizations, he focuses on further concepts that have been put forward and are somewhat related to technoscientization such as the concepts of biomedicalization, technoscientific identity and biosociality. He concludes that biomedicine, technoscience and rare disease patient organizations are interrelated in complex and heterogeneous ways, resulting in ambiguous situations of detectable, but also limited technoscientization of this particular field.

*Ulrich Fiedeler* focuses on the concept of technoscience as put forward by Weber (2010) before he presents a broader historical overview of the role of technology in modern science. Closely addressing the task to “probe technoscience,” he comes to a similarly ambiguous conclusion as Wehling, albeit with a different empirical focus—namely on modern physics and the emerging field of nanotechnology—and different arguments. He questions the thesis of a recent epochal break from science to technoscience, placing *the* major shift in the relation between nature and technique already in the sixteenth century. He consecutively interprets contemporary phenomena like nanotechnology as a renaissance of modern conceptions of science or as a result of gradual change that has already started centuries ago. But he also allows for the possibility that the situation might be different for the life sciences.

*Jan C. Schmidt* further intensifies the historical analysis by presenting an in-depth discussion of Francis Bacon’s science programme and programmatic. He probes the concept of technoscience by first delineating four different notions of technoscience, referring either to a difference in (1) motives, interests, purposes and power, in (2) method, practice, process and action, in (3) objectivity, evidence and truth or in (4) ontology and objects. He further adds that to subscribe to the notion of technoscience one does not have to subscribe to a difference in all of the four dimensions. After summarizing the peculiarities of technoscience as described by different contemporary authors, Schmidt provides an analysis of Bacon’s programme along the same four dimensions and concludes that Bacon should indeed be conceived as a forerunner of the same real-constructivist materialist epistemology that demarcates current technoscience.

*Karen Kastenhofer* and *Jan C. Schmidt* in their essay set out to further elaborate the conception of technoscience by re-constructing the different idea(l)s prevalent in science, technology and technoscience and their relation to the idea(l) of a powerful technoscience prevalent in science governance discourses from Francis Bacon to Vannevar Bush and current Initiatives. They start with the twofold programmatic presented in Hacking’s (1983) account of “Representing and Intervening” and—drawing on empirical studies of various epistemic cultures, such as the ones prevalent in ecological, biotechnological, synthetic and systems biological research—add two further idea(l)s about/of scientific practice. They enlist contemplative, interventionist, constructionist and creationist stances and see them invested with an orientational function when it comes to technoscientific research practices, making sense of research outcomes within technoscience and referring to technoscientific research (be it research outcomes, products or regulation) within society, thereby demarcating a technoscience era.

From a philosophical point of view, *Federica Timeto* concentrates on the epistemologies and ontologies put forward by important technoscience analysists like Karen Barad, Katherine Hayles and Donna Haraway. By closely delineating their approaches, Time to rather probes current epistemologies and ontologies and their aptness to depict core characteristics of technoscience than probing a presumed difference between science and technoscience or the prevalence of a technoscientization in fields of practice. Her essay thereby contributes to a topic also addressed in Schmidt’s notion of real-constructivism and the discussion of representation and intervention presented in the essay by Kastenhofer and Schmidt, but focuses on Hayle’s refined model of constrained constructivism, Barad’s theory of agential realism and intra-action and Haraway’s idea of diffraction.

Overall, the five papers included in this special issue share an attempt to probe the notion of technoscience. They approach this goal from different angles, in different ways and by different means—regarding the actual presence of technoscientization in a practical context like biomedicine and health care, the epochal break thesis announcing a new technoscience era, the relation between Bacon and contemporary technoscience, the demarcation of technoscience from science and technology, the relation between technoscience idea(l)s and technoscientific practice and the ontological conception of technoscience. Besides analyses of technoscience as a programme and/or practice, as a scientific and/or societal denominator, the five papers also touch upon socio-political issues, be it biosociality and illness identities (Wehling) or the governance of (powerful) technoscience (Kastenhofer and Schmidt). Taking up the notion of technoscience and demarcating “technoscience” from “normal” science can also be seen as a contribution to world making and hence a deeply political action. Hence, the now already long-running discussions about technoscience seem worth leading and the examples given in this essay help to illustrate the various implications linked to the technoscience discourse in various contexts.
